# *Mentha spicata*-mediated silver nanoparticles for combating *Streptococcus mutans* and oral cancer cells

**DOI:** 10.1038/s41598-025-23852-9

**Published:** 2025-11-04

**Authors:** Abdullah Yousef, Salem S. Salem, Ahmed Ragab, Omnia Mohamed El Shahaly, Habiba Omar Rateb, Reham Ahmed El-Esawy, Ahmed Mohamed Elakraa, Ehab S. Abd El Hamid, Sara Ibrahim

**Affiliations:** 1 Basic and Medical Sciences Department, Faculty of Dentistry, Al-Ryada University for Science and Technology, Sadat City, Menoufia Egypt; 2 Department of Botany and Microbiology, Faculty of Science, Al-Azhar University, Nasr City, Cairo 11884 Egypt; 3 Department of Chemistry, Faculty of Science Boys, Al-Azhar University, Nasr City, Cairo 11884 Egypt; 4 Chemistry Department, Faculty of Science, Galala University, Galala City, Suez 43511 Egypt; 5 Oral Pathology Department, Faculty of Dentistry, Ain Shams University, Cairo, Egypt

**Keywords:** Green synthesis, *M. spicata*, Biogenic nanoparticles, *S. mutans*, Biofilm inhibition, Anticancer activity, Biochemistry, Biotechnology, Cancer, Drug discovery, Microbiology, Nanoscience and technology, Plant sciences

## Abstract

A sustainable and ecologically safe substitute for traditional chemical synthesis, eco-friendly synthsis of silver nanoparticles (Ag-NPs) using plant-based extract offers promising biological opportunities. In this study, Ag-NPs were rapidly synthesized from an aqueous extract of *Mentha spicata* leaves and analyzed using a variety of techniques, including atomic force microscopy (AFM), transmission electron microscopy (TEM), zeta potential measurement, X-ray diffraction (XRD), UV–vis spectroscopy, and Fourier transform infrared spectroscopy (FTIR). Having a negative surface charge (− 32.5 mV) and an average width of 33 nm, the resultant nanoparticles were primarily spherical, indicating their colloidal stability. *Streptococcus mutans* ATCC 25175 was significantly inhibited by the antimicrobial evaluation, which revealed a minimum inhibitory concentration (MIC) of 0.125 mg/mL and a distinct inhibition zone of 20 mm. Planktonic growth was unaffected, however biofilm formation decreased by 77.3% (*p* < 0.05) at sub-MIC values (0.031 mg/mL). Computational docking further indicated potential interactions of Ag-NPs with dihydroorotase synthase and quorum sensing regulatory proteins, suggesting a mechanistic role in disrupting biofilm development. The nanoparticles also showed a high level of cytotoxicity against OECM-1 oral cancer cells, with an IC_50_ of 24.21 µg/mL and an inhibition of up to 91.6% at 62.5 µg/mL. Collectively, these outcomes underscore the dual antimicrobial and anticancer properties of *M. spicata*-derived Ag-NPs, positioning them as eco-friendly candidates for biomedical exploration.

## Introduction

Nanotechnology, according to the Environmental Protection Agency (EPA), is the study and manipulation of materials with sizes between one and one hundred nanometers^1,2^ . Smaller metals have a larger surface area than their bulk or non-nano counterparts when evaluated at the nanoscale^3,4^. Additionally, because of the tiny size effect, interface effect, interaction effect, and quantum effect, they display distinct physical and chemical properties not found in non-nano metals^5,6^. Numerous types of nanoscale metals are used widely in biology, medicine, and engineering^7–10^. For example, both Gram-positive and Gram-negative bacteria are inhibited in their development and activity by nanoparticles^11–13^. *Streptococcus mutans* is known to be a major cause of dental caries because of its ability to create biofilms and convert food carbohydrates into acids that gradually destroy enamel. Because of its clinical significance in oral infections and resistance to traditional antibiotic treatments, the standard strain of *S. mutans* ATCC 25175 (American Type Culture Collection, USA) was used in this investigation^14^. *Mentha spicata* is a well-known aromatic herb used in traditional medicine and the food industry. Its many phytochemicals are primarily responsible for its numerous pharmacological characteristics, which include antibacterial, antioxidant, anti-inflammatory, and anticancer effects. This plant contains a rich profile of polyphenols, flavonoids, terpenoids, and essential oils, with carvone and limonene being predominant bioactive compounds^15,16^. Owing to this phytochemical diversity, in the green manufacturing of metallic nanoparticles, *M. spicata* extracts have lately been investigated as natural, environmentally benign reducing and stabilizing agents^17^. Green chemistry and nanotechnology have enormous potential to provide new and necessary goods that benefit industry, the environment, and human health^18^. So, Green synthesis is a method of reducing metal ions that uses plant extracts rather than industrial chemical agents. Green synthesis is better than normal chemical production because it is less costly, emits fewer harmful emissions, and improves environmental and public safety^19^. Recent advancements in the manufacturing of inorganic nanoparticles, which comprise a range of nanomaterials, have demonstrated their potential for a wide range of uses^20,21^. Metal nanoparticles have become more viable options for antimicrobial interventions, offering the prospect of mitigating or eradicating the escalating prevalence of bacterial resistance. A significant proportion of these nanoparticles are composed of heavy metals, such as silver. In particular, silver nanoparticles have garnered widespread adoption in antimicrobial applications, demonstrating efficacy against antibiotic-resistant bacteria and those implicated in hospital-acquired infections^22,23^.

Biofilm formed by *S. mutans* in the oral cavity provide essential protection as complex communities encased within an extracellular matrix^24^. Serving as a physical barrier, these biofilm shield bacteria, enhance adhesion to tooth surfaces, and facilitate glucan synthesis from sucrose via glycosyltransferases. Interactions with salivary proteins reinforce biofilm formation and bacterial adhesion, while unique nutrient gradients promote bacterial survival and proliferation^25^. The potential of silver nanoparticles (Ag-NPs) to prevent bacterial biofilms and have antibacterial properties has been extensively studied^26–28^. Against *S. mutans*, these nanoparticles act through multiple mechanisms, including disruption of cell membranes, interference with extracellular matrix development, induction of reactive oxygen species (ROS), suppression of quorum sensing pathways, and controlled release of silver ions. Collectively, these actions compromise biofilm stability and lead to bacterial cell death^29^.

Quorum sensing and gene regulation mechanisms enable bacteria to adapt and resist antimicrobial stressors within biofilm. Additionally, matrix production by *S. mutans* contributes to biofilm stability and surface attachment. Understanding these mechanisms is vital for combating oral diseases associated with *S. mutans* biofilm^30^.

Serious side effects, pharmacokinetic limitations, and diagnostic difficulties are often the limits of conventional cancer therapy. There are intriguing ways to get around these problems with nanotechnology. Nanoparticles’ distinct physicochemical characteristics such as surface charge, nanoscale size, and structural adaptability have greatly improved cancer treatment and diagnosis methods^8^. Their application in nanomedicine has driven major progress in drug delivery, leading to highly specific systems that can accurately transport therapeutic agents to targeted cancer cells^31^.

**Fig. 1:**
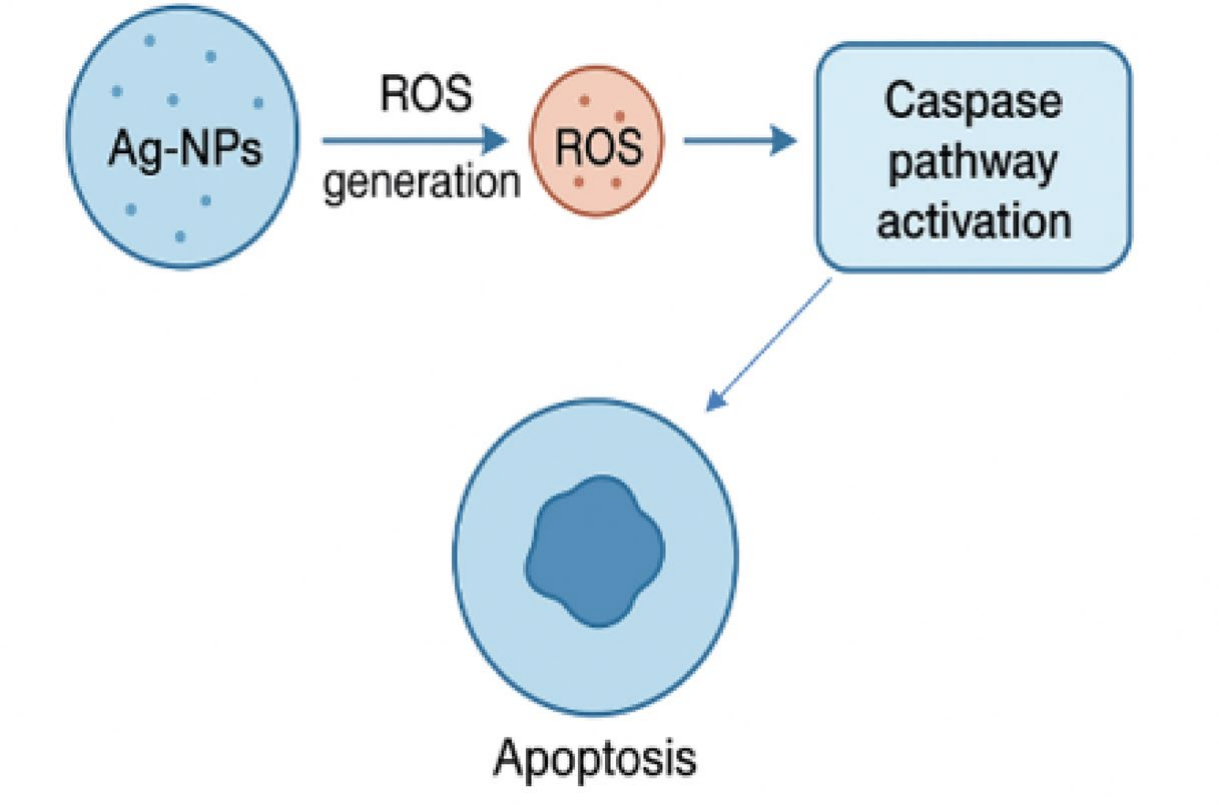
Proposed mechanism by which Ag-NPs induce apoptosis via ROS generation and caspase pathway activation.

A promising and economical method of treating cancer, the green production of nanotherapeutic agents utilizing elements obtained from plants offers benefits in terms of safety and therapeutic performance^32^. Researchers paid attention to silver among all the noble metals because of their availability and unique properties as nanomaterials^33,34^. After much research, it was found that Ag-NPs has inhibitory effects against cancerous cell line activities. According to cancer type, the suitable plant, metal (Ag, Cu, or other), shape, size, and IC_50_ of NPs is determined to have the best inhibitory effect^35^. Nanoparticles eliminate cancer cells through multiple pathways, with apoptosis (programmed cell death) being one of the most prominent. A key mechanism is the induction of reactive oxygen species (ROS), which generates oxidative stress that damages cellular membranes, nucleic acids, and proteins. This damage initiates the intrinsic apoptotic cascade, where caspases such as Caspase-9 and Caspase-3 are activated. Once triggered, these enzymes degrade essential cellular structures, resulting in the controlled death of the malignant cell, as illustrated in Fig. 1^36–38^. This study aims to evaluate the antibacterial and anticancer activities of silver nanoparticles synthesized using *Mentha spicata*, with a particular focus on their ability to inhibit *Streptococcus mutans* biofilm formation and exert cytotoxic effects against OECM-1 oral carcinoma cells.

## Materials and methods

### Plant material and authentication

In December 2024, *M. spicata* plants grown under cultivation were obtained from the Egyptian Ministry of Agriculture according to standard procedures and with official consent. Identification of the species was confirmed at the Department of Botany and Microbiology, Faculty of Science, Al-Azhar University. It was deposited in the department’s herbarium.

### Preparation of *M. spicata* leaves extract

Following thorough cleaning with distilled water, the collected leaves were ground into a fine powder and left to dry in the shade at 50 °C. Approximately 10 g of the powdered material were macerated in 150 milliliters of distilled water. Then, after two minutes of boiling (100 °C), it was allowed to cool to room temperature before being filtered through Whatman No. 1 filter paper. The resulting extract was kept in storage at 4 °C for use in further investigations.

### GC–MS analysis of *M. spicata* leaves extract

The extracts were analyzed for their chemical profile using a Focus GC-TSQ Evo8000 Mass Spectrometer (Thermo Scientific, Austin, TX, USA) fitted with a direct capillary TR-5MS column (30 m × 0.25 mm i.d., 0.25 μm film thickness). It began at 60 °C, increased by 5 °C per minute to 250 °C with a 2-minute hold, and then increased by 10 °C per minute to 300 °C. Using helium as the carrier gas, the injector temperature was set to 270 °C and the flow rate was maintained at 1 mL/min. After a two-minute solvent delay, 1 µL of the diluted extract was automatically injected in split mode using an AS3000 Auto-sampler. Electron ionization (EI) was performed in full-scan mode at 70 eV, scanning around the 50–550 mass-to-charge (m/z) range. The ion source and transfer line were maintained at 200 °C and 250 °C, respectively. To identify the chemicals, their retention times and mass spectra were compared to reference information from the WILEY 09 and NIST 14 libraries^39^.

### Synthesis of Ag-NPs

A 1 mM stock solution was made by dissolving 0.0169 g of silver nitrate (AgNO_3_) in 100 mL of distilled water after buying the substance from Sigma-Aldrich. For the green synthesis of nanoparticles, 50 mL of the AgNO₃ solution (1 mM) was gradually mixed with 5 mL of *M. spicata* leaf extract, and the mixture was then incubated at 20 °C without stirring. A color shift from clear to yellowish-brown, which finally turned dark brown, signaling nanoparticle stability, clearly verified the Ag-NPs synthesis development The reaction conditions were adjusted to pH 7.5, and optimization was carried out by altering the extract-to-metal ratio (1:10–1:20), incubation temperature (20–60 °C), and reaction duration (2–6 h) to yield nanoparticles with favorable size, morphology, and stability.

### Evaluation of Ag-NPs properties

The successful synthesis of Ag-NPs was confirmed by the reaction mixture’s observed color shift. To ensure thorough characterization, several analytical methods were applied. UV-Vis spectroscopy was used to assess the nanoparticles’ optical properties. Fourier Transform Infrared Spectroscopy (FTIR) was used to identify the functional biomolecules in the *M. spicata* extract and to investigate the surface interactions of the nanoparticles. Data on particle size distribution, surface charge, and colloidal stability were obtained using dynamic light scattering (DLS) and zeta potential analysis. Furthermore, the crystalline structure of the biosynthesized Ag-NPs was verified by X-ray diffraction (XRD), and the crystallite dimensions were determined using Scherrer’s equation:

$$D=\frac{{K\lambda }}{{\beta ~cos~\theta }}$$

where λ is the X-ray wavelength (1.5406 Å for Cu Kα radiation), β is the full width at half maximum (FWHM, in radians), θ is the Bragg diffraction angle, D is the crystallite size, and K is the form factor (0.9). Finally, Transmission Electron Microscopy (TEM) analysis allowed for the imaging of particle morphology and size distribution. Additionally, to do advanced atomic force microscopy (AFM) investigations and precisely ascertain the size and shape of the Ag NPs, a thin layer of Ag-NPs was applied on sterile, clean glass pieces that had been air-dried before analysis.

### Antibacterial activity

#### Test organisms

Using *S. mutans* ATCC 25175, a widely used reference strain from the American Type Culture Collection (ATCC, USA), Ag-NPs were evaluated for their antibacterial and antibiofilm properties.

#### Antibacterial assay

Diffusion technique using agar wells was employed to evaluate the Ag-NPs’ antibacterial efficacy.

Strains of bacteria 0.5 Mc Farland 1.3 × 10^8^ Colony Muller Hinton agar (MHA) medium was used to seed individual forming units (CFU) inoculum. Using a cup-borer (6.5 mm in diameter), a well was cemented after the media was produced in the plates. Ag-NPs (0.5 mg/mL) in 30 µL volumes were added to each well. The plates were then pre-diffused for two hours at 8°C in a refrigerator and then incubated for the whole night at 37 °C. The diameter of the inhibition zone was measured to determine the degree of bacterial growth inhibition^40^. Three runs of the experiments were conducted, and the mean values are shown.

### Minimum inhibitory concentration (MIC)

Finding the minimum inhibitory concentration (MIC) of Ag-NPs was done using the 96-well microtiter plate (MTP) method. To achieve a McFarland standard of 0.4 (≈ 1.2 × 10^8 CFU/mL), *S. mutans* ATCC 25175 was grew overnight in nutritious broth. By diluting this solution further in broth, the working inoculum was produced. The final capacity of each well was 200 µL, which was achieved by successively diluting 100 µL of bacterial culture with 100 µL of Ag-NPs solution. Positive control wells contained bacterial inoculum but no Ag-NPs, while negative controls included growth media with Ag-NPs but no bacterial inoculum. Each experiment was carried out three times, and the mean results were recorded. A microplate reader (Tecan Elx800, Fitchburg, WI, USA) was used to measure the bacterial growth at 620 nm following 18 to 24 h of incubation at 37 °C. The minimum inhibitory concentration, or MIC, was the lowest concentration of Ag-NPs that completely stopped detectable growth (lack of turbidity) when compared to the controls^41^.

### Anti-biofilm activity of Ag-NPs

The *S. mutans* ATCC 25175 strain was used to test Ag-NPs’ capacity to prevent biofilm development. The cells were able to form biofilms because the nanoparticles were added to the growing media now of inoculation. The 96-well microtiter plate (MTP) test was used to assess Ag-NPs’ antibiofilm efficacy against *S. mutans* ATCC 25175. In short, Ag-NPs were serially diluted twice using Trypticase soy broth supplemented with 1% glucose (TSB-Glc) (0.125–0.016 mg/mL; sub-MIC values). Bacterial suspensions were then added to each well until the final inoculum density was 5 × 10^5 CFU/mL. A microplate reader (Tecan Elx800, Fitchburg, WI, USA) was used to detect the optical density at 620 nm after a 24-hour incubation period at 37 °C in order to quantify the formation of biofilms. Biofilm inhibition was calculated by comparing wells treated with Ag-NPs to untreated controls. Each experiment was conducted three times, and the mean outcomes were reported^42^.

### Experimental section of molecular docking simulation

The Molecular Operating Environment (MOE) software, version 2014.0901, was used to perform the molecular docking investigations of Ag-NPs^43–45^. The Protein Data Bank provided the target proteins, dihydroorotase synthase (PDB ID: 2VEG) and quorum-sensing regulator (PDB ID: 4JVI), along with their 3D crystal structures (https://www.rcsb.org/structure/4VEG, accessed on 15 August 2024; https://www.rcsb.org/structure/2VEG, accessed on 25 January 2025). Using the method described by Ragab et al.^46^ and Basseem et al.^47^, Chain A was utilized to identify the active areas of both receptors. For molecular docking, the Triangle Matcher placement method employed London dG as the first rescoring function and GBVI/WSA dG as the second. Refining after docking was done using the Forcefield technique. To identify the optimal docking arrangement, the maximum binding affinity (lowest binding energy) was utilized. The Ag-NPs structural model was produced in accordance with previously published^47,48^.

### Anticancer activity

#### Cell culture

The OECM-1 oral cancer cell line was selected for this study. The cells were grown in F-12 K medium (Invitrogen, Karlsruhe, Germany) supplemented with 10% heat-inactivated fetal bovine serum (FBS) and 1% penicillin-streptomycin solution. A humidified incubator with 5% CO₂ and 37 °C was used to maintain the cultures^49^.

### MTT antitumor assay

The MTT assay was used to evaluate Ag-NPs’ cytotoxic effect. In this experiment, the hydrophilic yellow tetrazolium salt (MTT) is transformed into insoluble purple formazan crystals by the mitochondrial enzymes of viable cells; the intensity of these crystals is correlated with the vitality of the cells. The assay was conducted following previously described protocols. Briefly, OECM-1 cells were seeded in 96-well microtiter plates at a density of 1.5 × 10⁴ cells/mL in 100 µL of growth medium and incubated for 24 h. The next day, cells were filtered through 0.45 μm syringe filters to sterilize them after being treated in triplicate with Ag-NPs dissolved in 0.5% DMSO. Following the testing of concentrations between 0.4 and 100 µg/mL, the plates were incubated in 5% CO₂ at 37 °C for 24 h.After replacing the culture medium with a new media containing MTT solution, the plates were incubated for four hours at 37 °C in 5% CO₂. Following their dissolution in 100 µL of 1% HCl in isopropanol, the resulting formazan crystals were measured for absorbance at 570 nm using a microplate reader. The half-maximal inhibitory concentration (IC₅₀) was calculated by plotting a viability curve against Ag-NPs concentrations^50^.

Using the following formula, cell viability was calculated.

$${\text{cell viability }}\% =\left( {{\text{absorbance sample}}/{\text{ absorbance control}}} \right){\text{ }} \times {\text{ 1}}00.$$

### Statistical analysis

The values displayed are the averages of three independent, duplicate trials. The data was analyzed using a one-way ANOVA to ascertain the statistical significance of the group differences. After establishing a significance threshold of *p* < 0.05 for the analysis, a Tukey’s test was used to perform multiple comparisons to find differences.

## Results

### Characterization of *M. spicata* leaves extract using GC–mass analysis

**Table 1:** Major bioactive compounds identified by GC- mass in *M. spicata* leaves extract.

RT	Compound name	Area %	Molecular formula	Molecular weight
4.55	BICYCLO[3.1.1]HEPTANE, 6,6-DIMETHYL-2-METHYLENE-, (1 S)-	3.06	C_10_H_16_	136
5.53	Eucalyptol	8.41	C_10_H_18_O	154
10.54	Carvone	18.15	C_10_H_14_O	150
14.49	(-)-á-Bourbonene	5.24	C_15_H_24_	204
15.31	Caryophyllene	7.10	C_15_H_24_	204
16.33	á-copaene	4.08	C_15_H_24_	204
16.76	á-ylangene	3.95	C_15_H_24_	204
17.13	ç-Elemene	1.82	C_15_H_24_	204
17.33	BICYCLO[7.2.0]UNDEC-4-ENE, 4,11,11-TRIMETHYL-8-METHYLENE-, [1R-(1R*,4E,9 S*)]-	1.60	C_15_H_24_	204
17.63	trans-Calamenene	2.38	C_15_H_22_	202
18.94	6,9,12,15-Docosatetraenoic acid, methyl ester	1.59	C_23_H_38_O_2_	346
19.81	à-acorenol	1.49	C_15_H_26_O	222
20.65	cis-5,8,11,14,17-Eicosapentaenoic acid	1.45	C_20_H_30_O_2_	302
24.96	17-Octadecynoic acid	4.50	C_18_H_32_O_2_	280
25.80	13-Heptadecyn-1-ol	2.38	C_17_H_32_O	252
30.04	Phytol	24.17	C_20_H_40_O	296
34.68	Cholestan-3-ol, 2-methylene-, (3á,5à)-	1.40	C_28_H_48_O	400
36.73	8,11,14-Eicosatrienoic acid, (Z, Z,Z)-	1.16	C_20_H_34_O_2_	306
37.62	9,12,15-Octadecatrienoic acid, 2,3-dihydroxypropyl ester, (Z, Z,Z)-	2.03	C_21_H_36_O_4_	352
39.68	DOTRIACONTANE	4.03	C_32_H_66_	450

**Fig. 2:**
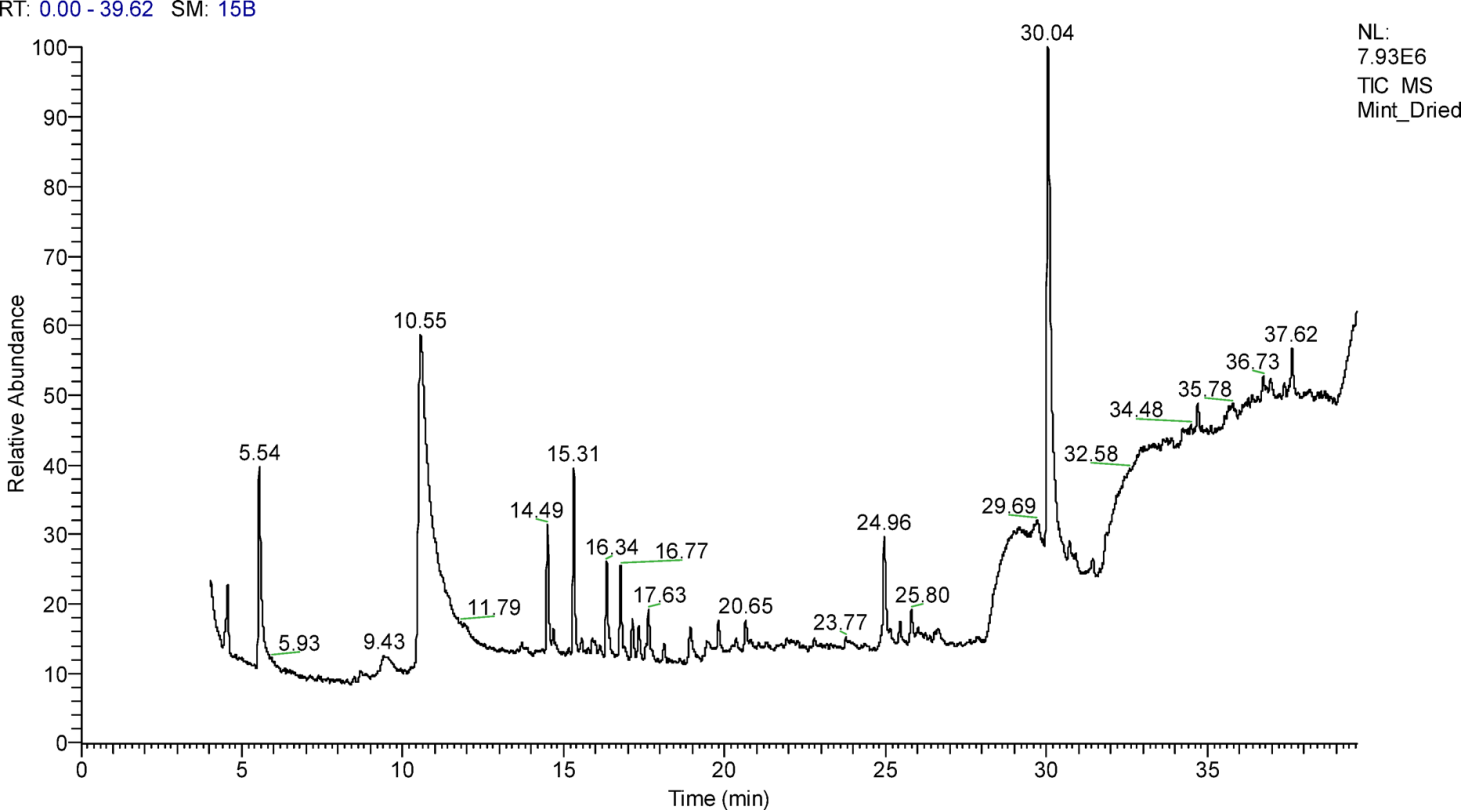
Major bioactive compounds identified by GC- mass in *M. spicata* leaves extract.

Characterization of *M. spicata* leaves extract. Figure 2; Table 1 illustrate the percentage of different compounds in prepared *M. spicata* leaves extract and represented 20 compounds. The main chemical components were Phytol 24.17%, Carvone 18.15%, Eucalyptol 8.41%.

### Preparation and characterization of Ag-NPs

**Fig. 3:**
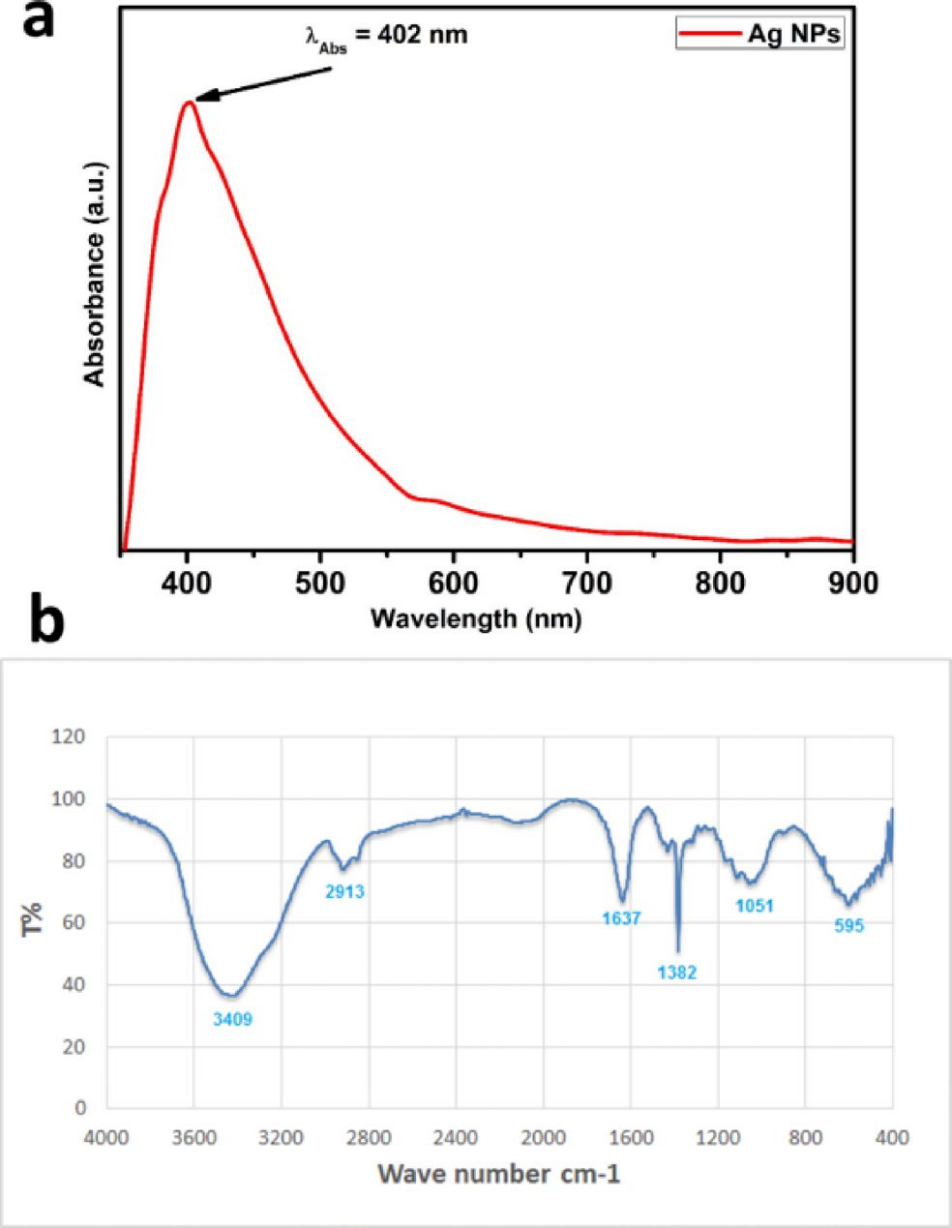
: The UV–vis spectrum (**a**) and FTIR spectra (**b**) for Ag-NPs produced by *M. spicata* leaf extract.

UV–vis absorption spectra Fig. 3a confirms that Ag-NPs drive surface plasmon oscillations inside the particles, giving them a light-yellow to brown color. confirming the formation of Ag-NPs from 402 nm. the incorporation. The as-prepared Ag-NPs produced in the presence of *M. spicata* leaf extract exhibit surface plasmon bands (SPR) with a characteristic wavelength range of 402 nm, validating the synthesis of Ag-NPs. The FTIR spectra of the *M. spicata* leaf extract display a few prominent peak areas at 3409, 2913, 1637, 1382, 1051, and 595 cm^− 1^ (Fig. 3b). During the biosynthesis of the silver nanoparticles, the phytochemicals present in the *M. spicata* leaf extract acted as reducing and capping agents. In the FTIR spectrum, discrete absorption peaks corresponded to different functional groups. It was determined that a broad band at 3409 cm⁻¹ was caused by the O–H stretching vibrations of amino acids, tannins, and phenols. These vibrations are mostly in charge of converting Ag⁺ ions to Ag⁰ and stabilizing the nanoparticles at the same time. The peak at 1051 cm⁻¹ correlated with the C–O stretching vibrations of organic acids and carbohydrates, but the absorption band at 1637 cm⁻¹ showed C =O stretching of carbonyl groups. By avoiding aggregation and preserving nanoscale dimensions, these biomolecules also helped to stabilize nanoparticles. The participation of various phytoconstituents in the synthesis and long-term stability of Ag-NPs is confirmed by the FTIR data taken together, as seen in Fig. 3b and Table 2 .

**Table 2:** FTIR peaks and functional groups.

Probable plant compound	Probable functional group	Peak (cm⁻¹)
Phenols, tannins, amino acids	O–H stretching (hydroxyl)	3409
Amino acids, carbohydrates	C–H stretching	2913
Amino acids, organic acids	C=O stretching (carbonyl)	1637
Amino acids, phenols	C–H bending (methyl/methylene)	1382
Phenols, organic acids, carbohydrates	C–O stretching	1051
Phenols, tannins	C–H bending (aromatic)	595

**Fig. 4:**
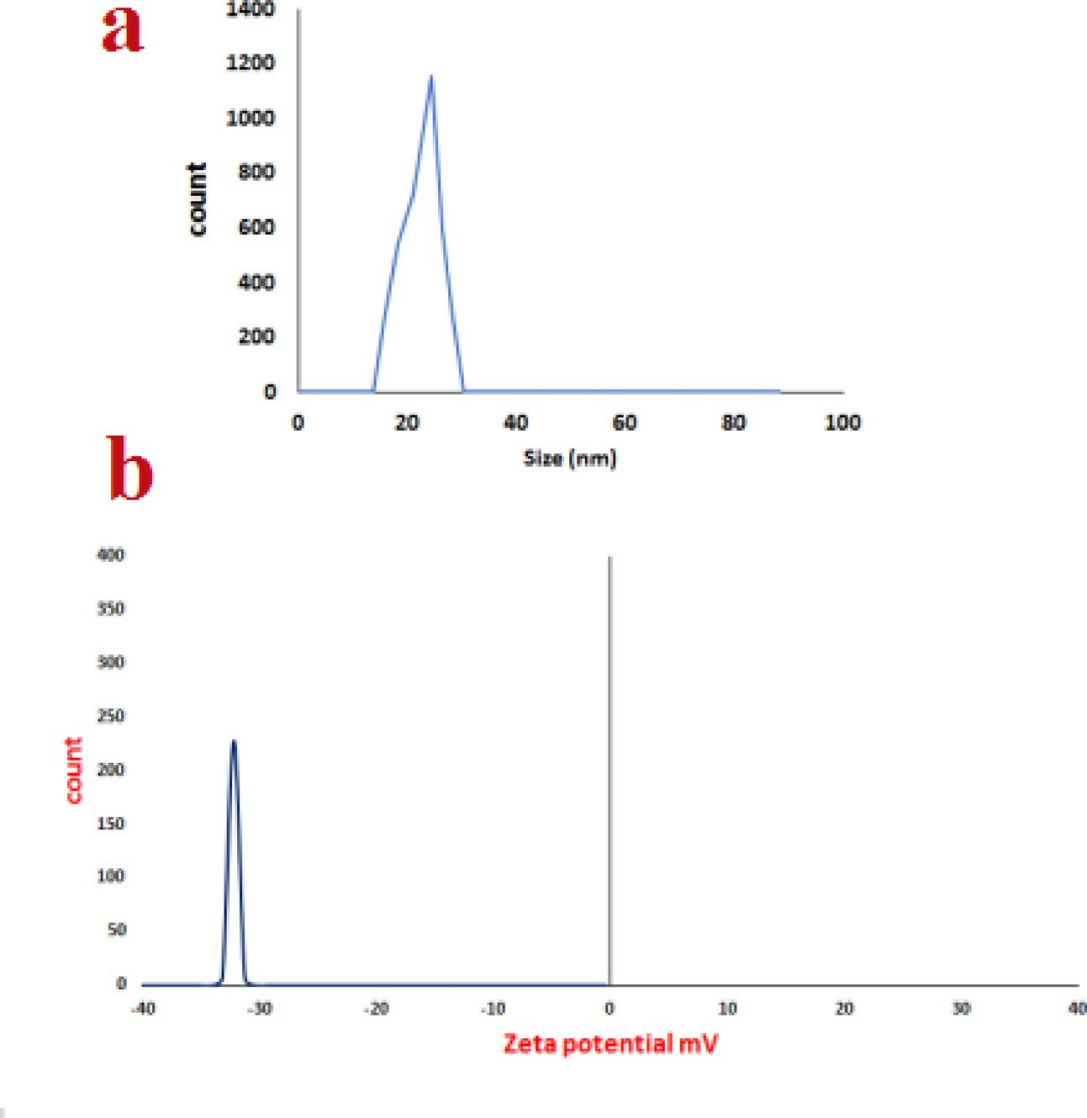
(**a**) Dynamic light scattering (DLS) analysis showing the size distribution of Ag-NPs. (**b**) Zeta-potential measurements indicating the surface charge and colloidal stability of the Ag-NPs.

Using dynamically scattered light (DLS) at different intervals and zeta potential methods, the surface and colloidal stability of the as-prepared Ag-NPs were investigated (Fig. 4). Ag-NPs’ hydrodynamic diameter (HD), or size, was clearly around 21 nm, and their polydispersity index (PDI) was roughly 0.3 (Fig. 4a). Furthermore, the zeta potential of Ag-NPs was as low as − 32. 5 mV. The findings (Fig. 4b) show that the Ag-NPs electrostatically attract particles in the solution to one another.

**Fig. 5:**
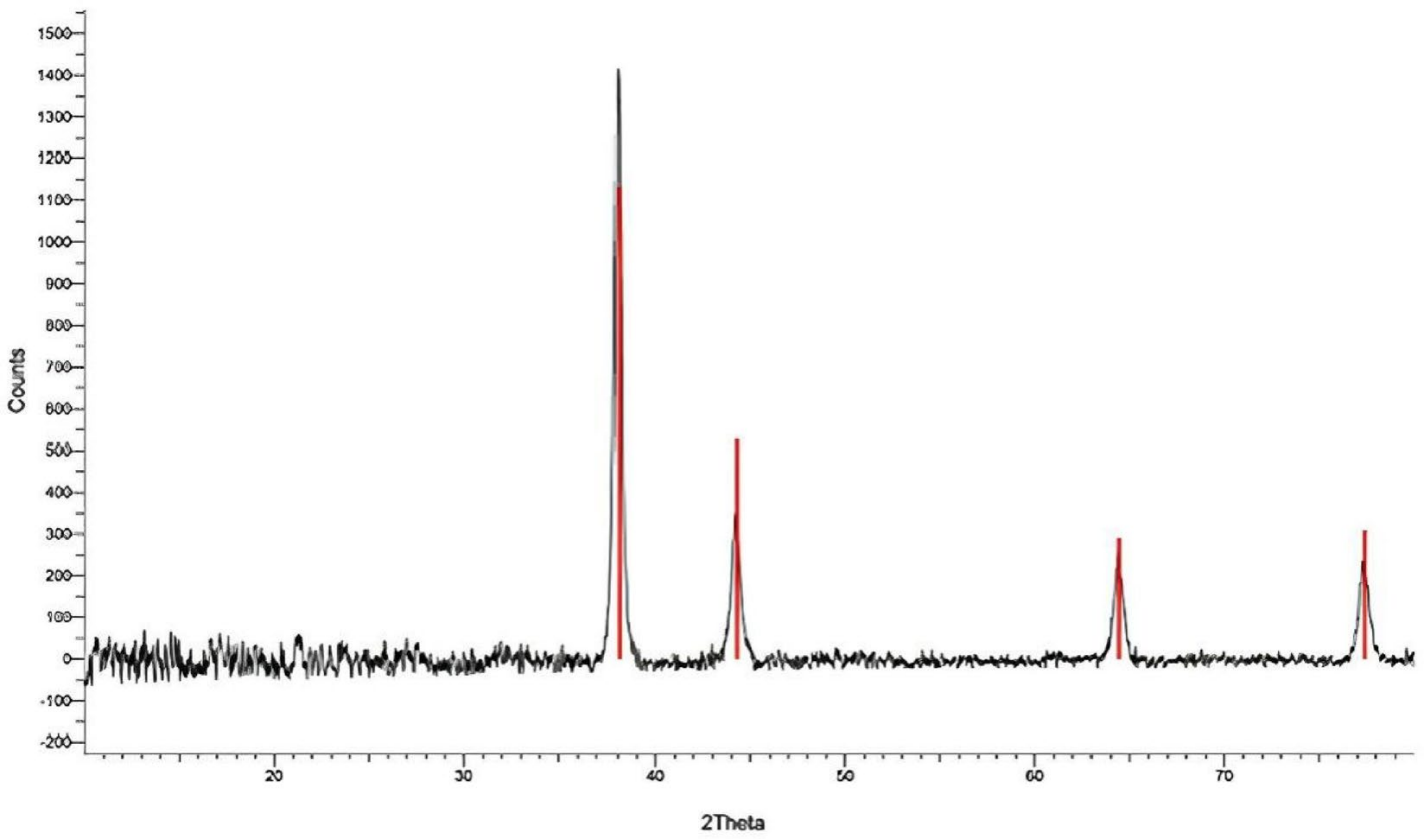
X-ray diffraction (XRD) pattern of Ag-NPs synthesized using *M. spicata* leaf extract, confirming their crystalline nature.

In this study, the crystallite size of the biosynthesized Ag-NPs was calculated from the principal diffraction peaks, as shown in Table 3; Fig. 5, using Scherrer’s equation. The calculated sizes ranged from 32 to 34 nm, with an average of about 33 nm. These results showed that the particle sizes were uniform in the (111), (200), (220), and (311) lattice planes. Additionally, the presence of clear and crisp diffraction peaks confirmed the high crystallinity and purity of the generated nanoparticles.

**Table 3:** Crystallite size of biosynthesized Ag-NPs calculated from the XRD diffraction peaks using scherrer’s equation.

Crystallite size D (nm)	FWHM (rad)	FWHM (°)	(hkl)	2θ (°)
33	0.00436	0.25	(111)	38.07
32	0.00471	0.27	(200)	44.2
34	0.00419	0.24	(220)	64.3
33	0.00454	0.26	(311)	77.2
33 ± 1 nm	Average			

**Fig. 6:**
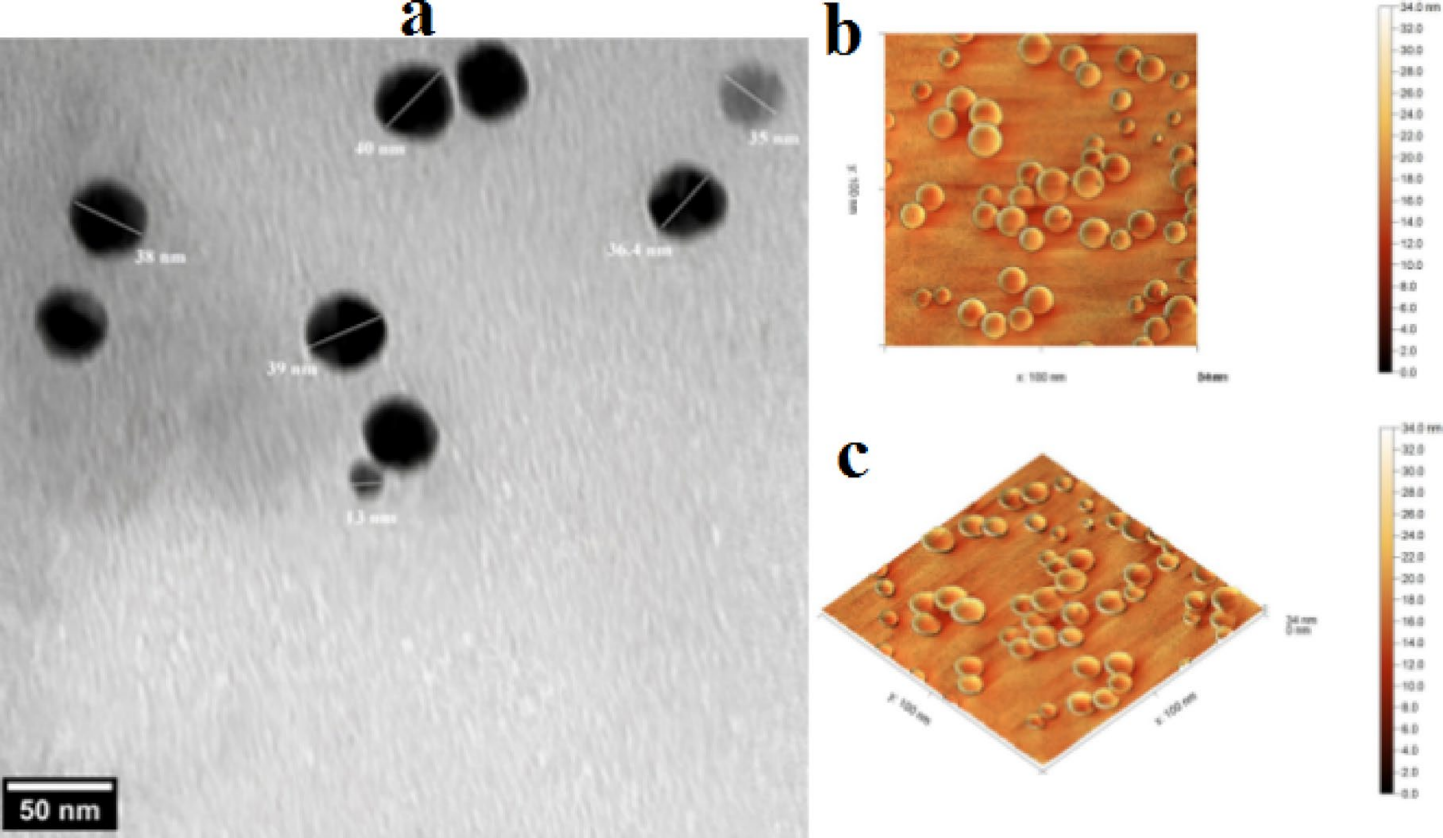
(**a**) Transmission electron microscopy (TEM) micrograph of Ag-NPs showing their morphology. (**b**) Two-dimensional atomic force microscopy (AFM) image of Ag-NPs. (**c**) Three-dimensional AFM image of Ag-NPs.

Figure 6 illustrates the Ag-NPs TEM picture. The nanoparticles’ average size was determined to be 33 nm, and Ag-NPs have one shape, all of which are sphere shape (Fig. 6a). Atomic force microscopy (AFM) provided two-dimensional and three-dimensional images of the Ag-NPs, which revealed that the nanoparticles were predominantly spherical in shape. The size distribution observed in the 2D and 3D images indicated an average diameter of approximately 17.5 nm (Fig. 6b and c).

### Antibacterial and MIC of Ag-NPs against *S. mutans*

**Fig. 7:**
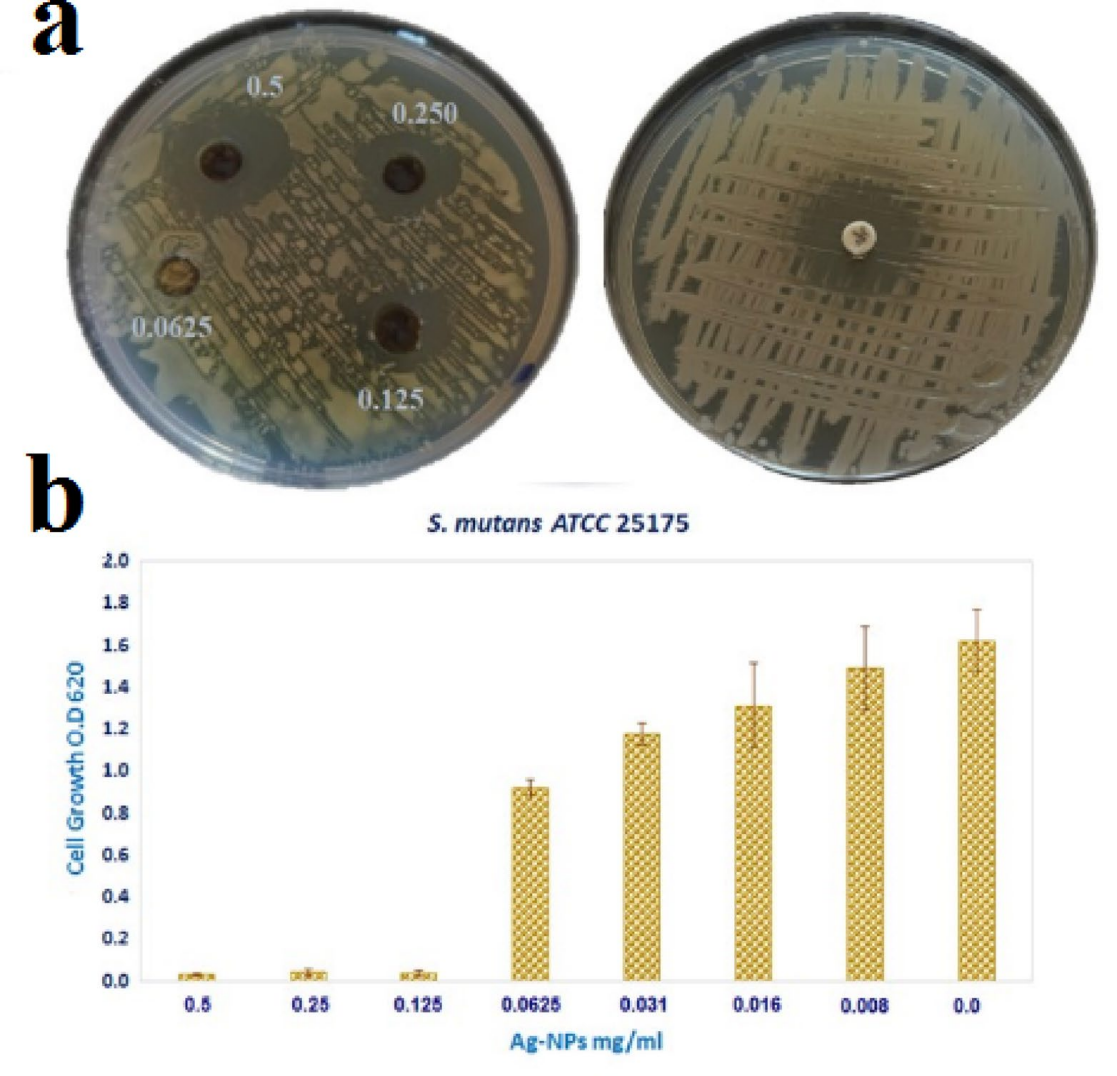
(**a**) The agar well diffusion method was used to measure the antibacterial activity of Ag-NPs against *S. mutans* ATCC 25175, (**b**) MIC of Ag-NPs against *S. mutans* ATCC 25175 assessed using the 96-well microtiter plate assay.

When tested against *S. mutans* ATCC 25175, the biosynthesized Ag-NPs showed strong antibacterial activity. A distinct inhibitory zone measuring 20 ± 1.2 mm was noted in the agar well diffusion assay. The MIC for the broth microdilution experiment was also found to be 0.125 mg/mL, which is like to the reference antibiotic levofloxacin (5 µg), as demonstrated in Fig. 7 .

### Effect of Ag-NPs on biofilm activity of *S. mutans*

**Fig. 8:**
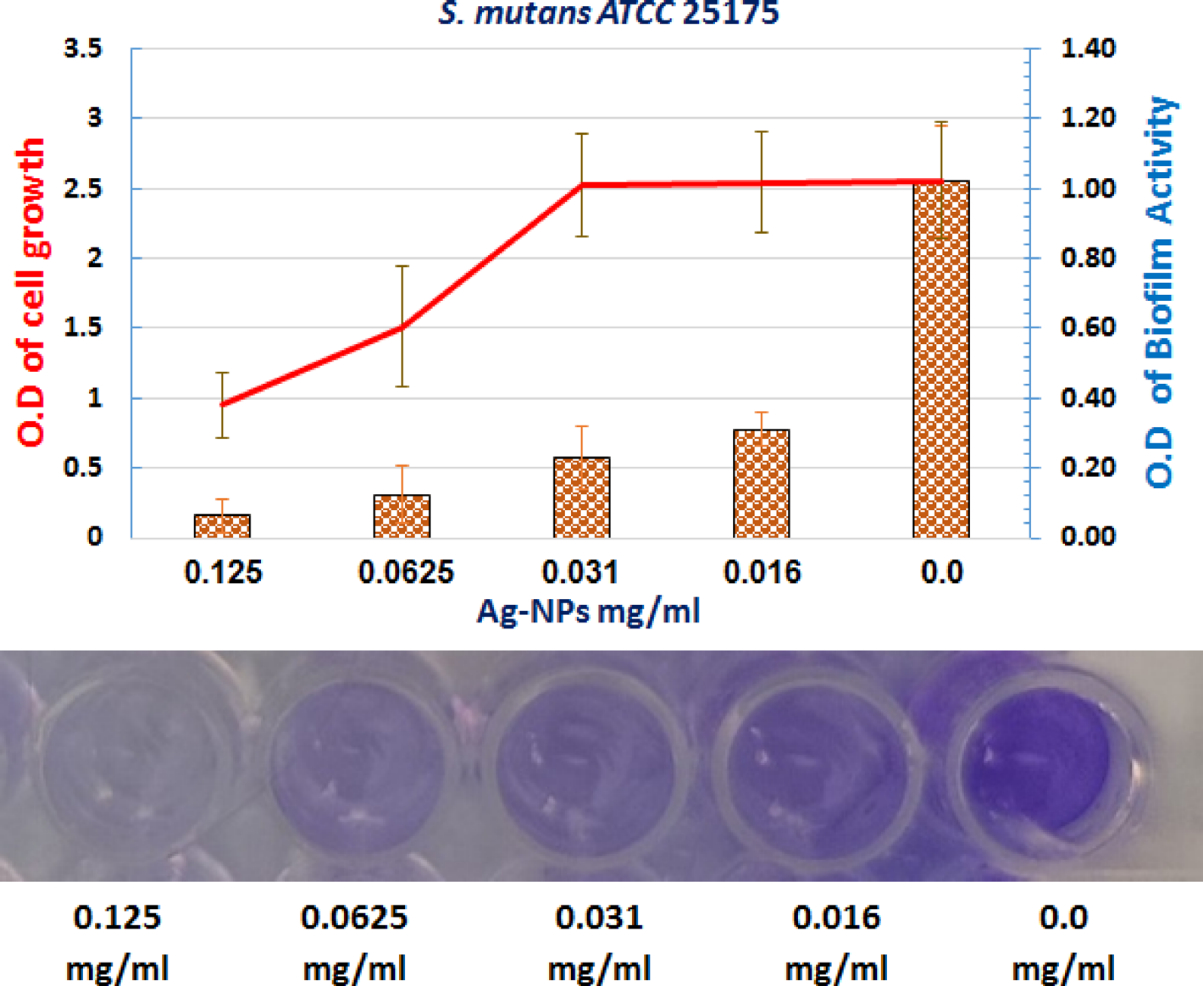
Effect of a sub-inhibitory concentration of Ag-NPs on the biofilm formation of *S. mutans*. The treatment resulted in a significant reduction in biofilm activity compared to the control.

Ag-NPs sub-inhibitory concentrations considerably reduced biofilm formation by *S. mutans*. At 0.031 mg/mL (1/4 MIC), biofilm biomass was reduced by 77.3% compared to untreated controls (*p* < 0.05) as shown in Table 4; Fig. 8.

**Table 4:** Anti-biofilm effect of sub-inhibitory dose of Ag-NP against *S. mutans*.

Concentration mg/mL	Ag-NPs		
	Biofilm activity		Growth of *S. Mutans* ATCC 25175
	OD 620	% of Reduction	OD 620
0.125	0.104 ± 0.05	89.81 ± 0.6	0.451 ± 0.23
0.0625	0.131 ± 0.08	87.85 ± 0.5	1.51 ± 0.43
0.031	0.231 ± 0.09	77.37 ± 0.7	2.52 ± 0.37
0.016	0.311 ± 0.05	69.54 ± 0.6	2.56 ± 0.36
0	1.021 ± 0.16	100 ± 1.6	2.54 ± 0.42

OD: optical density. %: percentage of biofilm activity.

### Docking simulation

**Fig. 9:**
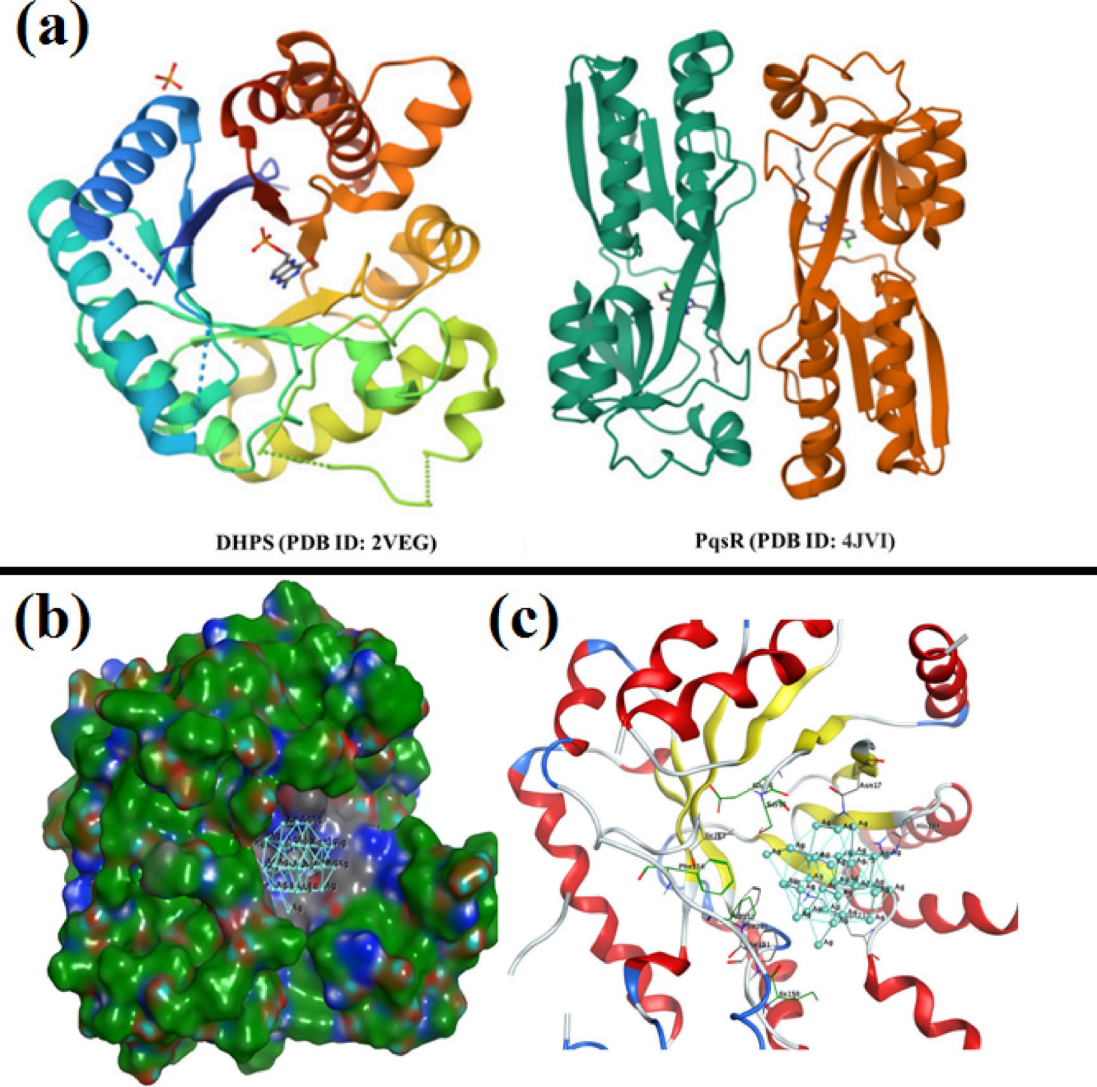
The protein data bank provided the 3D crystallography of the DHPS and PqsR enzymes attached to their co-crystallized ligands (**a**). The dihydroorotase synthase (DHPS; PDB: 2VEG) active site’s three-dimensional structure of Ag-NPs (turquoise) (**b**) displays the surface representation, whereas (**c**) the binding mode of Ag-NPs within the active site highlights interactions with amino acid residues.

To determine possible molecular targets underlying Ag-NPs’ antibacterial activity, molecular docking simulations were used in this study to assess the interaction of Ag-NPs with the active sites of dihydroorotase synthase (DHPS; PDB: 2VEG) and the quorum sensing regulator PqsR (PDB: 4JVI) Fig. 9a . The reliability of the docking approach was confirmed by the root mean square deviation (RMSD) values of 0.7813 Å for DHPS and 0.9216 Å for PqsR, which were obtained from the validation of both receptors using their co-crystallized ligands prior to docking. Ag-NPs demonstrated a binding energy of S = − 3.21 kcal/mol for DHPS (PDB: 2VEG) and created hydrophobic interactions with several amino acid residues inside the active site, such as Pro152, Ile150, Ser56, Phe151, Phe154, Ile112, Phe206, Asn17, and His284 (Fig. 9b and c).

**Fig. 10:**
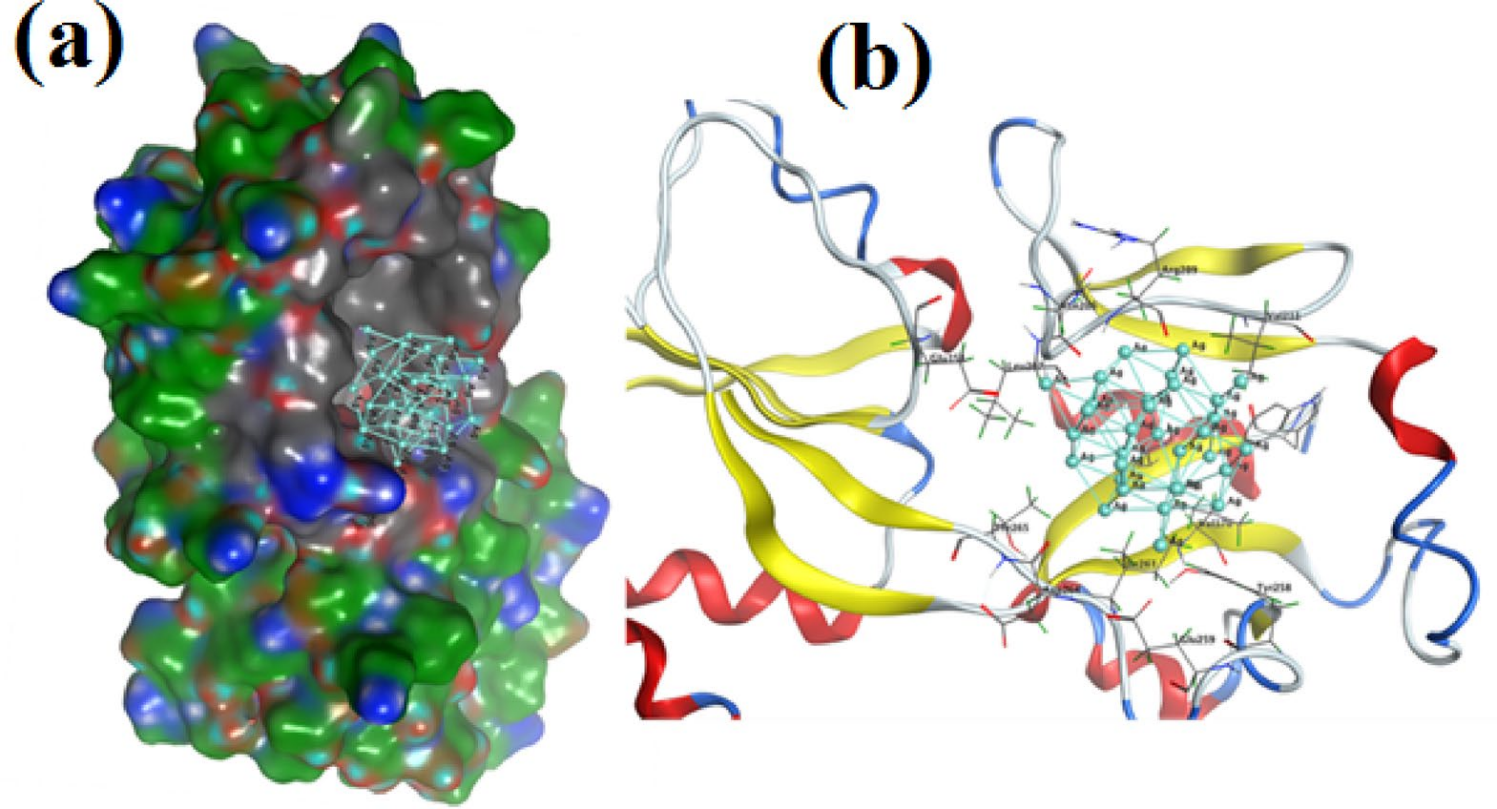
Ag-NPs (turquoise) in the quorum sensing regulator PqsR’s (PDB: 4JVI) active site: (**a**) Ag-NPs’ surface representation, (**b**) the binding mode of the Ag-NPs in the active site that highlights interactions with amino acid residues.

However, docking the Ag-NPs quorum sensing regulator (PqsR) (PDB: 4JVI) showed that the hydrophobic interaction between the pocket and silver lattice resulted in a binding energy S = − 2.94 kcal/mol. The amino acid residues Glu151, Leu207, Arg206, Val211, Ile263, Val170, Thr265, Asp264, Thr258, and Glu259 that reacted with Ag-NPs (Fig. 10a and b).

### Ag-NPs as a tumor-fighting agent utilizing MTT test

**Fig. 11:**
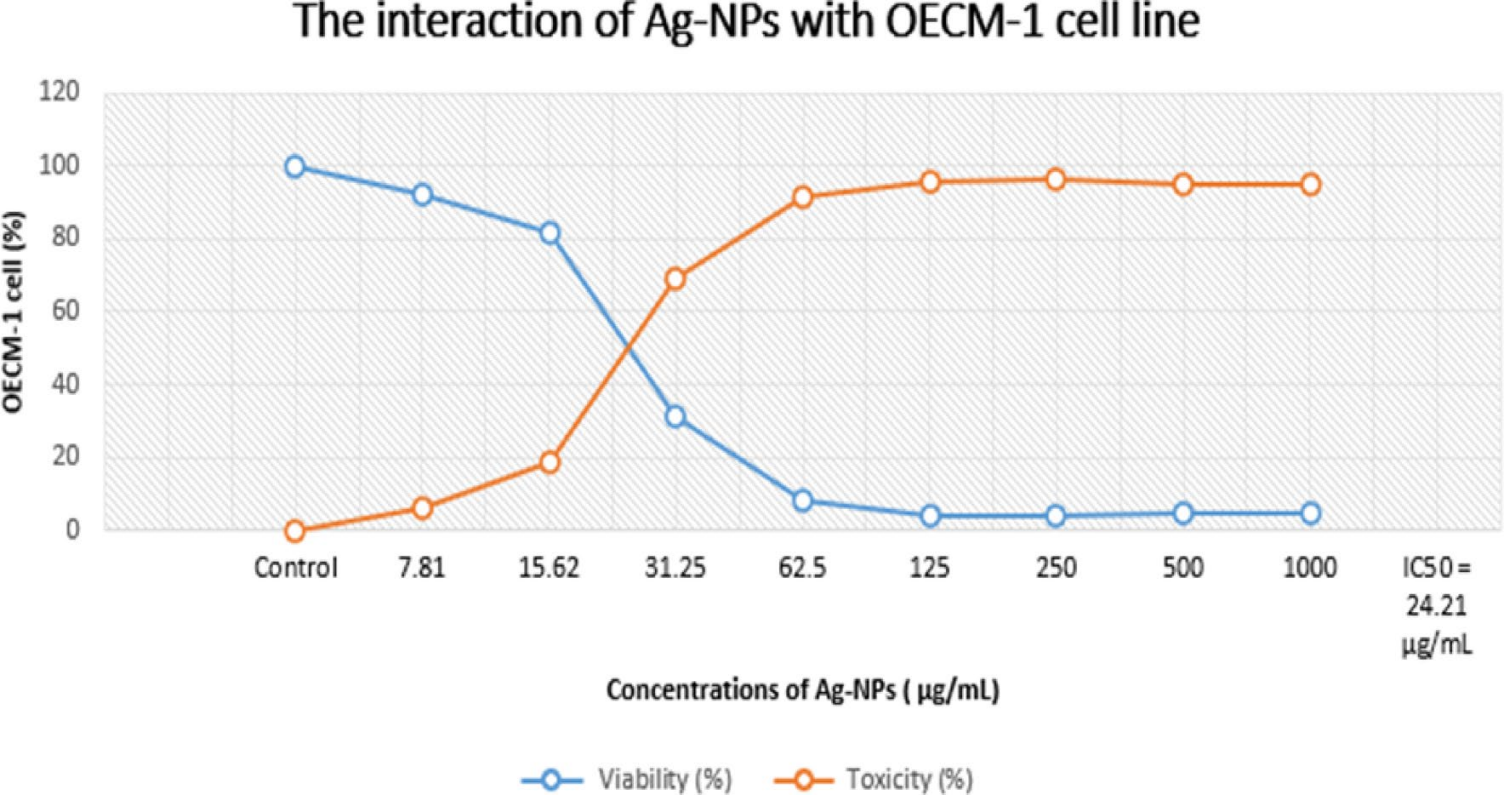
Interaction of Ag-NPs with the OECM-1 oral carcinoma cell line, demonstrating nanoparticle uptake and cellular response.

Table 5 provides a summary of Ag-NPs’ anticancer activity against OECM-1 cells. The dose-response curve was used to compute the IC₅₀ value, which is the concentration needed to inhibit 50% of cell viability. It came out to be 24.21 µg/mL. Using a range of Ag-NPs doses from 7.8 to 1000 µg/mL, the cytotoxic effect was assessed. Cell viability percentages at each concentration were 92.1, 81.5, 30.9, 8.4, 4.2, 3.9, 4.9, and 4.99%, respectively, demonstrating a dose-dependent reduction in OECM-1 cell viability, as stated in Fig. 11.

**Table 5:** The interaction of Ag-NPs with OECM-1 cell line.

	OECM-1 cell line	
Ag-NPs Conc. µg /mL	Viability (%)	Toxicity (%)
Control	100	0.0
7.81	92.1	5.99
15.62	81.5	18.5
31.25	30.9	69.1
62.5	8.4	91.6
125	4.2	95.8
250	3.9	96.1
500	4.9	95.1
1000	4.99	95.01
IC50 = 24.21 µg/mL		

## Discussion

In this study, a green synthesis process and a plant extract (such leaf extract from *M. spicata*) were used to create Ag-NPs. These results were consistent with previously published research by Chinnasamy et al.^51^. The phytochemical analysis of *M. spicata* leaf extract confirmed the presence of tannins, phenolics, flavonoids, alkaloids, terpenoids, and saponins. These bioactive compounds can serve as organic capping and reducing agents during the nanoparticle synthesis process due to their well-known reducing and antioxidant properties. Twenty chemicals were found in the extract by GC-MS analysis, with the main ingredients being Phytol (24.17%), Carvone (18.15%), and Eucalyptol (8.41%). While the ketone functional group of carvone helps to cap and stabilize nanoparticles, The hydroxyl and carbonyl groups of phytol and eucalyptol most likely contribute to the reduction of Ag⁺ ions to Ag⁰, hence reducing aggregation. These phytochemicals work together to produce Ag-NPs in an environmentally sustainable manner, increasing the stability of the nanoparticles over time^52,53^.

The biomolecules involved in the capping, reduction, and stability of Ag-NPs were identified using FTIR spectroscopy. Their involvement in the bioreduction of Ag⁺ ions is suggested by the presence of several functional groups. The functional groups in charge of nanoparticle generation and stability were revealed by comparing the band strengths in the spectra of the biosynthesized Ag-NPs and the leaf extract from *M. spicata*. This demonstrates that functional groups clearly play a part in the way forms are formed and arranged^54–56^. The formation of Ag-NPs was confirmed by UV–Vis spectroscopy, showing a characteristic surface plasmon resonance (SPR) peak at λmax = 402 nm. This value is consistent with previously reported plant-mediated Ag-NPs synthesized using *Combretum indicum*, which showed SPR peak at 405 nm^57^. The close agreement with these studies confirms the successful synthesis of stable Ag-NPs in our work. The stability of the nanoparticles was further assessed over 30 days, with negligible shift in λmax or change in color, indicating excellent colloidal stability. These findings of DLS and Zeta were consistent with one published earlier by Yousef et al.^58^. The X-Ray diffractometer in this work verified that the biologically produced Ag-NPs nanoparticles were crystalline. This was accomplished by continuously scanning the equipment from a wide range of angles. Similar Ag-NPs peaks were also observed by other researchers in the XRD profile^22,59,60^.

The crystallite size of the biosynthesized Ag-NPs was approximately 33 nm, based on Scherrer’s equation. This amount is consistent with previous reports on plant-mediated Ag-NPs, where crystallite sizes were 35 nm^61^, confirming the reliability and reproducibility of the green synthesis process. The biological activity of Ag-NPs, including their cytotoxic and antibacterial qualities, is mostly determined by the surface-to-volume ratio, which is increased by their nanoscale dimensions. *M. spicata* leaf extract is a powerful natural reducing and stabilizing agent that guarantees high crystallinity and consistent nanoparticle production in the environmentally friendly synthesis of Ag-NPs. This is further supported by the agreement between our results and those of other investigations. AFM measurements highlight the exterior texture and topologies of green produced Ag-NPs, demonstrating their spherical shape. These results were consistent with previous studies by Daphedar and Taranath^62^. In the present study, Ag-NPs synthesized using *M. spicata* leaf extract exhibited significant antimicrobial activity against *S. mutans* ATCC 25175. The antibacterial potential of plant-mediated Ag-NPs against oral pathogens has been demonstrated in several previous in vitro studies, highlighting their promise as alternative therapeutic agents^63^. For example, Ag-NPs produced from Justicia glauca leaves exhibited antibacterial activity against *S. mutans*,* Lactobacillus acidophilus* (*L. acidophilus*), and *Staphylococcus aureus* (*S. aureus*)^64^. Furthermore, plant extracts from *Salvadora persica*, *Ficus bengalensis*, and *Azadirachta indica* showed antibacterial activity against *Lactococcus lactis* (*L. lactis*), *L. acidophilus*, and *S. mutans*. According to^63^, MIC of 0.2 mg/mL and an inhibitory zone of 18.30 ± 0.5 nm, biogenic Ag-NPs from gum arabic shown antibacterial activity against *S. mutans*. In another investigation, this was found^64^ also noted that Ag-NPs’ MIC against *S. mutans* was 0.28 mg/mL. According to Selvaraj et al.^65^, a dose of 0.016 mg/mL of TA-AuNPs completely eradicated preformed *S. mutans* biofilm. The mechanism through which Ag-NPs exerts its inhibitory impact on pathogens is the ability of Ag-NPs to change the resistant species’ cell walls and membranes, likely as a result of the creation of pores on the cell wall and membrane^59,66^.

Numerous studies indicate that the antibacterial activity of Ag ions is mostly due to their positive charge, which facilitates electrostatic interactions between the negatively charged bacterial cell membranes and the positively charged nanoparticles^23,67^. A crucial computational method for forecasting the three-dimensional configurations of protein–ligand complexes and clarifying their molecular interactions is molecular docking simulation^68^. Moreover, the docking simulation for nanomaterials can help us better understand reaction mechanisms and suggest possible modes of action The enhanced antibacterial and antibiofilm activities observed for Ag-NPs can be partly explained by their interactions with specific bacterial proteins. Molecular docking studies showed that Ag-NPs potentially interact with dihydropteroate synthase (DHPS, PDB ID: 2VEG), an enzyme essential for folic acid biosynthesis, and PqsR (PDB ID: 4JVI), a quorum sensing regulator protein. DHPS shares functional similarity with *S. mutans*, making it a relevant target, while PqsR-related quorum sensing pathways in *S. mutans* are involved in biofilm formation. These interactions may underline the observed inhibition of biofilm formation and bacterial growth, providing mechanistic insight that supports our experimental findings.

Ag-NPs’ antibacterial properties are ascribed to their capacity to block the regulatory mechanisms for quorum sensing and dihydroorotase synthase (DHPS). Ag-NPs have a greater affinity for DHPS than for quorum sensing regulators, according to binding energy studies, and their activity is mostly mediated by hydrophobic contacts inside the active site at distances smaller than 4.5 Å. Since cancer is still one of the most deadly illnesses in the world, new treatment approaches must be developed constantly. Because of their intrinsic cytotoxic qualities, metallic nanoparticles (MNPs) have garnered a lot of interest lately as potential anticancer treatments. Their programmability minimizes interactions with healthy cells and increases the potential for precision oncology by enabling focused medication delivery and selective detection of malignant cells^69,70^.

It has been shown that ecologically benign, biocompatible nanoparticles have been produced. They can facilitate a more efficient interaction with life systems because of this action. As a result of the many characteristics that nanoparticles contain, the detrimental impacts that they have are effective. There are a number of properties that are included in this category, including surface chemistry, concentration, shape, and size^71,72^. Ag-NPs use a variety of biological processes to produce their anticancer effects. Reactive oxygen species (ROS) are produced as one of the main mechanisms of action, upsetting the redox balance in cancer cells and causing oxidative stress-induced death. Overproduction of ROS harms cellular organelles, especially the mitochondria, causing cytochrome c release, activation of caspase-dependent apoptotic pathways, and loss of mitochondrial membrane potential. Furthermore, Ag-NPs can directly bind with DNA and proteins within cells, resulting in structural damage as well as disruption of transcription and replication^37^.

Modification of signaling pathways linked to cell survival and proliferation is another approach. According to earlier research, Ag-NPs tip the scales in favor of programmed cell death by upregulating pro-apoptotic proteins like Bax and downregulating anti-apoptotic proteins like Bcl-2. Additionally, Ag-NPs may stop the growth of cancer cells by causing cell cycle arrest at checkpoints (such as G0/G1 or G2/M)^36^. The enhanced permeability and retention (EPR) effect in tumor tissues and the increased metabolic activity of cancer cells may be the reasons for Ag-NPs’ selective cytotoxicity against cancer cells as opposed to normal cells. When considered collectively, these results imply that Ag-NPs produced using *M. spicata* not only stop the proliferation of OECM-1 oral cancer cells but also cause apoptosis by damaging mitochondria and disrupting apoptotic signaling pathways. These findings align with earlier research on the anticancer potential of Ag-NPs mediated by plants^73^.

Additionally, the small dimension and coating charge enhanced dispersion and mobility within the cancerous tissue^74^. Additional research has demonstrated that a liver tumor cell line was considerably cytotoxically impacted by Ag-NPs produced using *T. purpurogenus* external pigment^75^. HepG2 and MCF-7 cells were cytotoxically affected by Ag-NPs in a dosage-related manner^76,77^. In a different research, Ag-NPs were tested versus Caco2 at dosages varying from 1.953 to 4000 µg/mL. After a 24-hour exposure time, their efficacy against tumor had an IC_50_ value of 252.83 µg/mL^78^.

## Conclusion

In conclusion, Ag-NPs were successfully produced utilizing *M. spicata* leaf extract in a rapid and eco-friendly manner. The biosynthesized Ag-NPs’ spherical average size of 33 nm and negative surface charge of -32.5 mV verified their structural integrity. Even at sub-inhibitory doses, they demonstrated strong antibacterial action against *S. mutans*, successfully preventing the formation of biofilms. Additionally, Ag-NPs demonstrated significant cytotoxicity against OECM-1 cancer cells, suppressing proliferation up to 91.6% at 62.5 µg/mL and having an IC_50_ of 24.21 µg/mL. Molecular docking studies suggest that these biological effects may include interactions with dihydroorotase synthase and quorum sensing regulatory proteins. *M. spicata*-mediated Ag-NPs are appealing ecologically acceptable choices for biomedical applications due to their stability, nanoscale size, and combination antibacterial and anticancer qualities.

## Data Availability

The datasets generated during and/or analyzed during the current study are available from the corresponding author on reasonable request.
